# Supplementary material for: End Sequence Analysis Toolkit (ESAT) expands the extractable information from single-cell RNA-seq data
Source: Genome Res. 2016 Oct;26(10):1397–410. doi: 10.1101/gr.207902.116 (PMC5052061; doi:10.1101/gr.207902.116)
Supplement: Supplemental Material [file supp_gr.207902.116_Supp_Materials.tar.gz › ESAT-esat_v0.1/doc/html/classumms_1_1core_1_1model_1_1score_1_1_count_score.html]

ESAT: umms.core.model.score.CountScore Class Reference


ESAT


- Main Page
- Related Pages
- Packages
- Classes
- Files

- Class List
- Class Index
- Class Hierarchy
- Class Members

All Classes Namespaces Files Functions Variables Enumerator Pages

- umms
- core
- model
- score
- CountScore

Classes |
Public Member Functions |
Static Public Member Functions |
Static Public Attributes |
List of all members

umms.core.model.score.CountScore Class Reference

Inheritance diagram for umms.core.model.score.CountScore:

[legend]

Collaboration diagram for umms.core.model.score.CountScore:

[legend]

|  |  |
| --- | --- |
| Classes | |
| class | **Factory** |
|  | |
| class | **Processor** |
|  | |

|  |  |
| --- | --- |
| Public Member Functions | |
|  | CountScore (Annotation a) |
|  | |
|  | CountScore (CountScore other) |
|  | |
|  | CountScore (CountScore other, Annotation newAnnotation, double newCount) |
|  | |
|  | CountScore (AnnotationCollection<?extends Annotation > model, Annotation annotation) |
|  | |
|  | CountScore (AnnotationCollection<?extends Annotation > model, Annotation annotation, double regionTotal) |
|  | |
|  | CountScore (AnnotationCollection<?extends Annotation > model, Annotation annotation, boolean fullyContained) |
|  | |
|  | CountScore (AnnotationCollection<?extends Annotation > model, Annotation annotation, double regionTotal, boolean fullyContained) |
|  | |
|  | CountScore (Annotation annotation, double count, double regionTotal, double total) |
|  | |
| double | getCount () |
|  | |
| double | getScore () |
|  | |
| double | getTotal () |
|  | |
| double | getRegionTotal () |
|  | |
| double | getRPKM () |
|  | |
| double | getRPK () |
|  | |
| void | setCount (double count) |
|  | |
| void | setTotal (double total) |
|  | |
| void | setRegionTotal (double regionTotal) |
|  | |
| String | toString () |
|  | |
| boolean | equals (Object o) |
|  | |
| int | compareTo (CountScore o) |
|  | |
| int | hashCode () |
|  | |
| void | refreshPvalue () |
|  | |
| double | getPvalue () |
|  | |
| Public Member Functions inherited from umms.core.model.score.WindowScore.AbstractWindowScore | |
|  | AbstractWindowScore (Annotation t) |
|  | |
| Annotation | getAnnotation () |
|  | |

|  |  |
| --- | --- |
| Static Public Member Functions | |
| static double | asRPKM (double count, double total, int windowSize) |
|  | |

|  |  |
| --- | --- |
| Static Public Attributes | |
| static double | DEFAULT\_REGION\_TOTAL = -1.0 |
|  | |

|  |  |
| --- | --- |
| Additional Inherited Members | |
| Protected Attributes inherited from umms.core.model.score.WindowScore.AbstractWindowScore | |
| Annotation | annotation |
|  | |

## Detailed Description

Author
:   engreitz This class represents a simple count scoring function for the DataAlignmentModel.

## Constructor & Destructor Documentation

|  |  |  |  |  |  |
| --- | --- | --- | --- | --- | --- |
| umms.core.model.score.CountScore.CountScore | ( | Annotation | *a* | ) |  |

|  |  |  |  |  |  |
| --- | --- | --- | --- | --- | --- |
| umms.core.model.score.CountScore.CountScore | ( | CountScore | *other* | ) |  |

Here is the call graph for this function:

|  |  |  |  |
| --- | --- | --- | --- |
| umms.core.model.score.CountScore.CountScore | ( | CountScore | *other*, |
|  |  | Annotation | *newAnnotation*, |
|  |  | double | *newCount* |
|  | ) |  |  |

|  |  |  |  |
| --- | --- | --- | --- |
| umms.core.model.score.CountScore.CountScore | ( | AnnotationCollection<?extends Annotation > | *model*, |
|  |  | Annotation | *annotation* |
|  | ) |  |  |

Parameters
:   |  |  |
    | --- | --- |
    | model | Alignment data |
    | annotation | Region |

|  |  |  |  |
| --- | --- | --- | --- |
| umms.core.model.score.CountScore.CountScore | ( | AnnotationCollection<?extends Annotation > | *model*, |
|  |  | Annotation | *annotation*, |
|  |  | double | *regionTotal* |
|  | ) |  |  |

Parameters
:   |  |  |
    | --- | --- |
    | model | Alignment data |
    | annotation | Region |
    | regionTotal | Override region total |

|  |  |  |  |
| --- | --- | --- | --- |
| umms.core.model.score.CountScore.CountScore | ( | AnnotationCollection<?extends Annotation > | *model*, |
|  |  | Annotation | *annotation*, |
|  |  | boolean | *fullyContained* |
|  | ) |  |  |

Parameters
:   |  |  |
    | --- | --- |
    | model | Alignment data |
    | annotation | Region |
    | fullyContained | Only count fully contained reads |

Here is the call graph for this function:

|  |  |  |  |
| --- | --- | --- | --- |
| umms.core.model.score.CountScore.CountScore | ( | AnnotationCollection<?extends Annotation > | *model*, |
|  |  | Annotation | *annotation*, |
|  |  | double | *regionTotal*, |
|  |  | boolean | *fullyContained* |
|  | ) |  |  |

Here is the call graph for this function:

|  |  |  |  |
| --- | --- | --- | --- |
| umms.core.model.score.CountScore.CountScore | ( | Annotation | *annotation*, |
|  |  | double | *count*, |
|  |  | double | *regionTotal*, |
|  |  | double | *total* |
|  | ) |  |  |

Here is the call graph for this function:

## Member Function Documentation

|  |  |  |  |
| --- | --- | --- | --- |
| static double umms.core.model.score.CountScore.asRPKM | ( | double | *count*, |
|  |  | double | *total*, |
|  |  | int | *windowSize* |
|  | ) |  |  |

static

Here is the caller graph for this function:

|  |  |  |  |  |  |
| --- | --- | --- | --- | --- | --- |
| int umms.core.model.score.CountScore.compareTo | ( | CountScore | *o* | ) |  |

First compare counts Then compare annotations

Here is the call graph for this function:

|  |  |  |  |  |  |
| --- | --- | --- | --- | --- | --- |
| boolean umms.core.model.score.CountScore.equals | ( | Object | *o* | ) |  |

True iff count and annotation are equal

Here is the call graph for this function:

Here is the caller graph for this function:

|  |  |  |  |  |
| --- | --- | --- | --- | --- |
| double umms.core.model.score.CountScore.getCount | ( |  | ) |  |

Here is the caller graph for this function:

|  |  |  |  |  |
| --- | --- | --- | --- | --- |
| double umms.core.model.score.CountScore.getPvalue | ( |  | ) |  |

Here is the caller graph for this function:

|  |  |  |  |  |
| --- | --- | --- | --- | --- |
| double umms.core.model.score.CountScore.getRegionTotal | ( |  | ) |  |

Here is the caller graph for this function:

|  |  |  |  |  |
| --- | --- | --- | --- | --- |
| double umms.core.model.score.CountScore.getRPK | ( |  | ) |  |

Here is the call graph for this function:

Here is the caller graph for this function:

|  |  |  |  |  |
| --- | --- | --- | --- | --- |
| double umms.core.model.score.CountScore.getRPKM | ( |  | ) |  |

Here is the call graph for this function:

Here is the caller graph for this function:

|  |  |  |  |  |
| --- | --- | --- | --- | --- |
| double umms.core.model.score.CountScore.getScore | ( |  | ) |  |

Returns the number of reads overlapping this region

Returns
:   number of reds overlapping this window Returns the "score" as defined by each subclass (e.g. count, ratio, sum of annotation scores, etc.)

Implements umms.core.model.score.WindowScore.

Here is the call graph for this function:

Here is the caller graph for this function:

|  |  |  |  |  |
| --- | --- | --- | --- | --- |
| double umms.core.model.score.CountScore.getTotal | ( |  | ) |  |

Here is the caller graph for this function:

|  |  |  |  |  |
| --- | --- | --- | --- | --- |
| int umms.core.model.score.CountScore.hashCode | ( |  | ) |  |

|  |  |  |  |  |
| --- | --- | --- | --- | --- |
| void umms.core.model.score.CountScore.refreshPvalue | ( |  | ) |  |

|  |  |  |  |  |  |
| --- | --- | --- | --- | --- | --- |
| void umms.core.model.score.CountScore.setCount | ( | double | *count* | ) |  |

Here is the caller graph for this function:

|  |  |  |  |  |  |
| --- | --- | --- | --- | --- | --- |
| void umms.core.model.score.CountScore.setRegionTotal | ( | double | *regionTotal* | ) |  |

Here is the caller graph for this function:

|  |  |  |  |  |  |
| --- | --- | --- | --- | --- | --- |
| void umms.core.model.score.CountScore.setTotal | ( | double | *total* | ) |  |

Here is the caller graph for this function:

|  |  |  |  |  |
| --- | --- | --- | --- | --- |
| String umms.core.model.score.CountScore.toString | ( |  | ) |  |

Here is the call graph for this function:

## Member Data Documentation

double umms.core.model.score.CountScore.DEFAULT\_REGION\_TOTAL = -1.0

static

---

The documentation for this class was generated from the following file:

- java/umms/core/model/score/CountScore.java


---

Generated on Fri Aug 15 2014 15:11:12 for ESAT by  

 1.8.7
